# Linkages and key factors between soil bacterial and fungal communities along an altitudinal gradient of different slopes on mount Segrila, Tibet, China

**DOI:** 10.3389/fmicb.2022.1024198

**Published:** 2022-10-28

**Authors:** Tiantian Ma, Xinjun Zhang, Ruihong Wang, Rui Liu, Xiaoming Shao, Ji Li, Yuquan Wei

**Affiliations:** ^1^Institute of Tibet Plateau Ecology, Tibet Agricultural and Animal Husbandry University, Key Laboratory of Forest Ecology in Tibet Plateau, Ministry of Education, Nyingchi, Tibet, China; ^2^College of Resources and Environmental Science, Beijing Key Laboratory of Biodiversity and Organic Farming, China Agricultural University, Beijing, China; ^3^Organic Recycling Institute (Suzhou) of China Agricultural University, Wuzhong District, Suzhou, China

**Keywords:** soil microbial communities, Tibetan Plateau, elevational gradient, slope, microbial interaction, environmental influencing factor

## Abstract

Soil microbes are of great significance to many energy flow and material circulation processes in alpine forest ecosystems. The distribution pattern of soil microbial community along altitudinal gradients is an essential research topic for the Tibetan Plateau. Yet our understanding of linkages between soil microbial communities and key factors along an altitudinal gradient of different slopes remains limited. Here, the diversity, composition and interaction of bacterial and fungal communities and in response to environmental factors were compared across five elevation sites (3,500 m, 3,700 m, 3,900 m, 4,100 m, 4,300 m) on the eastern and western slopes of Mount Segrila, by using Illumina MiSeq sequencing. Our results showed that microbial community composition and diversity were distinct at different elevations, being mainly influenced by soil total nitrogen and carbonate. Structural equation models indicated that elevation had a greater influence than slope upon the soil microbial community. Co-occurrence network analysis suggested that fungi were stable but bacteria contributed more to among interactions of bacterial and fungal communities. Ascomycota was identified as a key hub for the internal interactions of microbial community, which might affect the soil microbial co-occurrence network resilience of alpine forest ecosystems on the Tibetan Plateau.

## Introduction

Soil microbial communities have an important role in energy flow and material circulation processes in terrestrial ecosystem, whose diversity can be used to characterize many aspects of the soil environment (Zhang et al., 2018). Bacterial and fungal communities has received much interest over recent decades with a view to identifying key biotic drivers of soil ecosystem services (Rashid et al., 2016). For example, bacteria and fungi as decomposers contribute to the regulation of organic matter and nutrient cycling, but have diverse substrate preferences and metabolic strategies, leading to distinctive organic carbon accumulation and stabilization outcomes in soils (Soares and Rousk, 2019). Bacteria and fungi usually share same microhabitats in soil but may have divergent ecological functions. Both bacterial and fungal communities are sensitive to the impacts from environmental disturbances entailing both biotic and abiotic factors, such as temperature, water content, pH, etc., which may in turn influence ecosystem stability (de Menezes et al., 2017; Wei et al., 2018). Therefore, focusing on co-occurring bacteria and fungi in soil as well as their interactions in response to varied environmental conditions could contribute to a more complete understanding of soil microbes’ respective roles.

The Tibetan Plateau, as the “third pole of world,” is crucial for global biodiversity and climate change due to the complex interactions there among environmental, cryospheric, and geographic processes (Wang et al., 2020). Abundant forestry resources can be found in the southeastern part of the Tibetan Plateau where alpine forest ecosystems fulfill an important role in maintaining the water balance, carbon sequestration, and even ecological security (Kai et al., 2016). Given the high average altitude, large elevation span, and seasonal freezing and thawing events of alpine forests in Tibetan Plateau, pronounced fluctuation patterns along elevational gradients and their associated environmental conditions may contribute differentially to microbial diversity and activity (Yue et al., 2016). Hence, obtaining a better understanding of the complex interactions among bacterial, fungal, and abiotic components in alpine forest soils is one of most challenging issues due to harsh geographic and climatic conditions, but is likely crucial to predict the stability of microbial networks’ response to future climate change (de Vries et al., 2018).

Distribution patterns of microbial communities along elevational gradients are an essential topic in biogeography, especially for mountains in the Tibetan Plateau, which have steeply compressed gradients in terms of abiotic and biotic conditions across short geographic distances (Bragazza et al., 2015; Yao et al., 2017). It is widely reported that the diversity and composition of soil microorganisms will vary across differing elevations (Bahram et al., 2012; Siles and Margesin, 2016; Ren et al., 2018). For example, work by Yang et al. (2014) indicated that the diversity of soil microbial genes in grasslands changes along an elevation gradient on the Tibetan Plateau. Klimek et al. (2015) found that soil bacterial catabolic activity in Beskidy Mountains was mainly affected by organic matter and dissolved organic nitrogen. Furthermore, autotrophic microbial composition in grassland soils on the Tibetan Plateau can markedly change along an elevational gradient and is jointly affected by temperature, nutrients, moisture, and plant types (Guo et al., 2015). A part of studies found that there is no general altitudinal pattern in bacterial diversity, and fungal biodiversity changed by ecological processes such as temperature along forest altitude gradients (Tian et al., 2017; Wang et al., 2017). Some studies showed that aboveground community composition and soil moisture played determining roles in restoring microbial community (Xu et al., 2014; Duan et al., 2021). Considering that altitude-driven environmental conditions on differing slopes of mountains may represent an analogue to ecological changes in different latitudinal-driven climatic zones (Diaz et al., 2003), that different variables act as key factors determining microbial community variation in mountains may be due to differing elevation ranges and neglecting the orientation of mountain slopes. In temperate regions of Asia, east- and south-facing mountain slopes may favor plant or microbial richness due to less cold temperatures, in comparison with western and eastern slopes of same mountains, especially in high altitude areas (Huang et al., 2015; Kumar et al., 2019). However, research on elevational patterns of microbial community structure and microbial interactions in alpine forest ecosystems on different slopes of mountains above 3,500 m a.s.l. remains rather limited.

In this study, Mount Segrila, located in the center area of a pristine forest tract in southeastern Tibet (Li et al., 2014), was selected to investigate the spatial patterning of bacterial and fungal community composition and diversity in soils at high elevation, ranging from 3,500 to 4,300 m a.s.l. Mount Segrila has a discernible and relatively geologically uniform altitude gradient, harboring vegetation representative of typical alpine forest on the Tibetan Plateau (Xu et al., 2014). Here, we used the Illumina MiSeq platform to compare the patterns of microbial communities at five elevations (3,500 m, 3,700 m, 3,900 m, 4,100 m, 4,300 m) on the eastern and western slopes of Mount Segrila. This study had three objectives: (1) to compare the diversity and composition of bacterial and fungal communities along an elevational gradient on different slopes; (2) to understand relationships between biotic and abiotic factors across elevations and between slopes; (3) to detect linkages of bacterial and fungal communities to key factors affecting the modes of microbial interactions.

## Materials and methods

### Site description and soil sampling

This study was carried out at Mount Segrila (29°10′ N–30°15′ N, 93°12′ E–95°35′ E), situated in Linzhi County, Tibet, China. Harboring typical montane frigid-temperate forest of southeastern Tibet, Mount Segrila has a peak elevation is >5,200 m a.s.l. and stark differences in climate and terrain between its east and west slopes. The climate here is humid and cool, with a mean annual temperature of −0.73°C, mean annual precipitation of 866 mm (mainly falling from April to October), and an annual evaporation of 544 mm (Xu et al., 2014). Soil and vegetation types in the studied elevational range are as follows: dark-brown forest soil (luvisols) from 3,500 to 4,200 m, with frigid, dark coniferous forests; black mattic soil (cambisols) from 4,200 to 4,500 m, with alpine sub-frigid shrub meadows; mainly forest between 2,700 and 4,300 m consisting of the conifer *Abies georgei* var. *smithii*. Shrubs such as *Sabina saltuaria* are dominant at higher elevations of 4,300–4,500 m.

**Figure 1:**
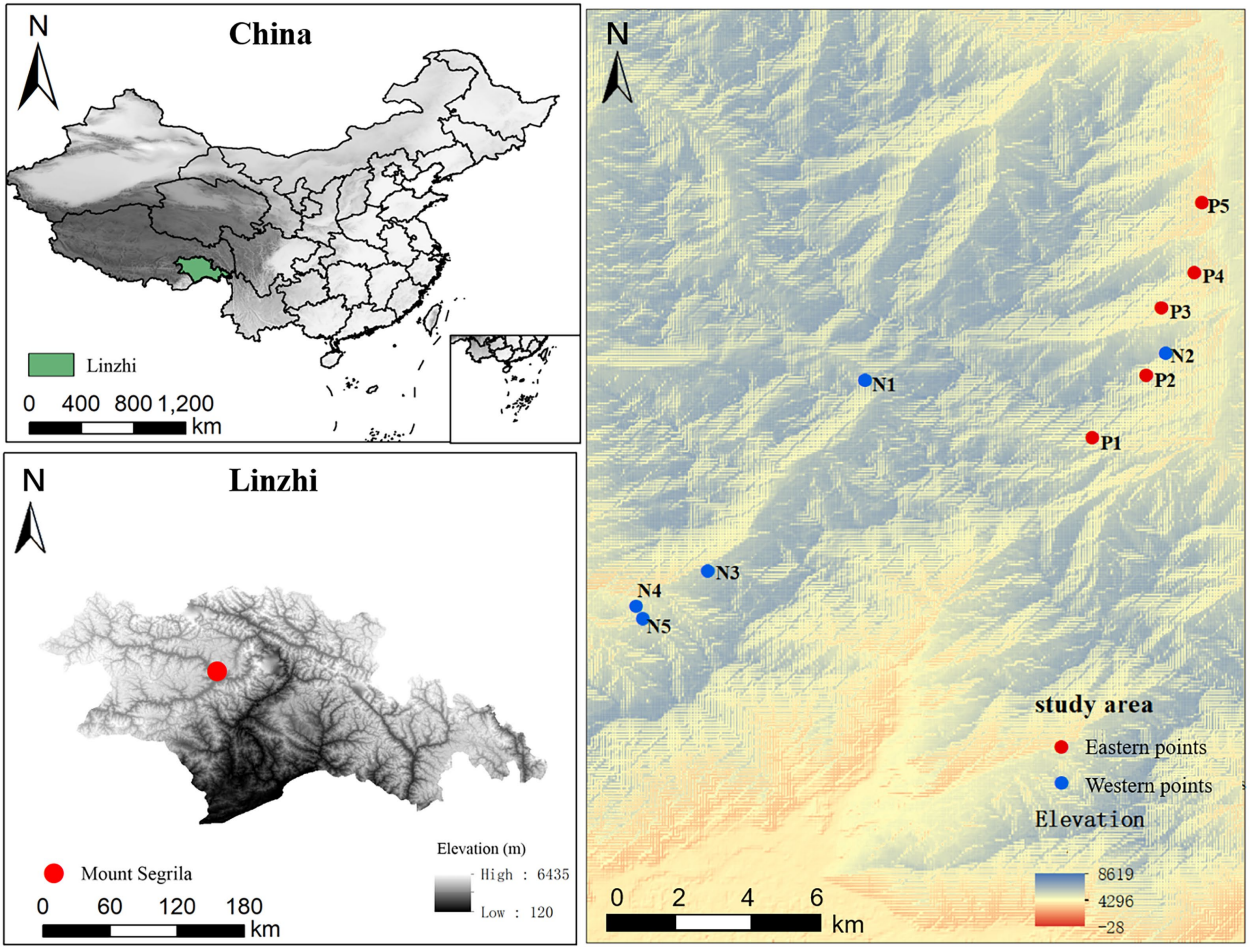
A map of the investigated area showing the locations of sampling sites along the altitudinal gradient on Mount Segrila, Tibet, China. P and N represent east slope and west slope, respectively. Different numbers mean different elevations (Number 1, 2, 3, 4, and 5 represent 4,300 m, 4,100 m, 3,900 m, 3,700 m, and 3,500 m, respectively).

Soil samples were collected from the five elevations (3,500 m, 3,700 m, 3,900 m, 4,100 m, and 4,300 m) of Mount Segrila, on both its east slope and west slope, in August 2020 (Figure 1). Soil samples were collected from nearby forest (within a 30-m distance). Fresh surface soil samples (each 20 cm × 20 cm, 0–15 cm in depth) were obtained after carefully removing the litter layer from each quadrat (*n* = 3 replicates per elevation site). Each quadrat’s soil sample consisted of mix of five subsamples randomly taken within a 10-m radius. All samples were passed through a 2.0-mm sieve to remove visible stones, invertebrate animals, large root fragments, and plant materials. One part of every sample was stored in a refrigerator at −80°C for DNA extractions, and the other part air-dried at room temperature to analyze the soil physical and chemical properties.

### Soil physicochemical and microbial analysis

Various soil physicochemical characteristics were determined, namely total organic carbon (TOC), total nitrogen (TN), C/N ratio, available phosphorus (AP), available potassium (AK), carbonate content (CO_3_^2−^), and pH. Soil pH was measured in a water-to-soil mass ratio of 5:1, using a pH meter. TOC and TN were measured with an elemental analyzer (Model EA1108, Carlo Erba, Torino, Italy; Wei et al., 2019). Soil AP was extracted with 0.5 M NaHCO_3_ and assayed by applying the ascorbic acid/molybdate reagent blue color method (Wei et al., 2018). Soil water content was quantified as the mass lost after drying at 105°C for 24 h. To determine the AK content, the ammonium acetate extraction-flame photometric method was used (Li et al., 2014); for carbonate content, it was determined by measuring the volume of CO_2_ gas released after applying an HCl treatment to the soil (Luo et al., 2015). Soil mechanical composition was assessed according to methodology of the Chinese Agricultural Industry Standard (NY/T 1121.3–2006).

Soil DNA was extracted using a FastDNA Spin Kit for soil (MP, Biomedicals, Santa Ana, Carlsbad, CA, United States), by following the manufacturer’s instructions. Purified DNA from each sample was PCR-amplified with the primer pair 338F (5′-ACTCCTACGGGAGGCAGCAG-3′) and 806R (5′-GGACTACHVGGGTWTCTAAT-3′) for the bacterial 16S rRNA gene, and likewise by the primer pair ITS1F (5′-CTTGGTCATTTAGAGGAAGTAA-3′) and ITS2R (5′-GCTGCGTTCTTCATCGATGC-3′) for the fungal ITS1 region, on an ABI GeneAmp^®^ 9,700 PCR thermocycler (ABI, CA, United States). The ensuing PCR products were collected using AxyPrep DNA Gel Extraction Kit (Axygen Biosciences, Union City, CA, United States). Purified amplicons were pooled in equimolar amounts and then paired-end sequenced on an Illumina MiSeq PE300 platform/NovaSeq PE250 platform (Illumina, San Diego, United States) by the Majorbio Bio-Pharm Technology Co. Ltd. (Shanghai, China), this done according to standard protocols. All obtained raw reads were deposited into NCBI Sequence Read Archive (SRA) database (accession number: SRP331508). After their sequencing, the operational taxonomic unit (OTU) assignment of the reads was carried out using >97% as the shared identity threshold, by the USEARCH (version 8.0.1623) from which representative sequences of OTUs were assigned for taxonomic analysis.

### Statistical analysis

Descriptive statistics including the mean, standard deviation (SD), minimum and maximum values, and the coefficient of variation (CV) were calculated in MS Excel software. Two-way analyses of variance (ANOVA) and multiple comparison tests (LSD [least significant difference]; all-pairwise comparisons test) were used to compare differences for different variables and test significance of differences (*p* < 0.05). For these statistical analyses, we used SPSS v 25.0 software (SPSS Inc., Chicago, IL). Data for physical and chemical indicators were using Origin 2021 software.

To express taxonomic alpha diversity, the Chao1, Shannon, and heip indexes were calculated in QIIME 2. Principal coordinate analysis (PCoA) was employed to graphically explore differences in microbial community structure between different elevation sites, based on Bray–Curtis distances using OTUs dataset, using Canoco v5.0. Redundancy analysis (RDA) with forward selection was also carried out in Canoco v5.0 software, to analyze the relationships between microorganisms and environmental factors. A “robust” maximum likelihood evaluation program was used to analyze a fitted structural equation model (SEM), in AMOS 24.0 software, based on several criteria: Chi-square (*p* > 0.05), high goodness-of-fit index (GFI > 0.90), and root mean square errors of approximation (RMSEA <0.05).

Microbial co-occurrence networks of mixed fungal and bacterial communities were constructed based on the relative abundances of OTUs. For this, the most abundant species (i.e., top 10%) in the bacterial and fungal communities were selected, that is 860 spp. of bacteria, 323 spp. of fungi. We used the MENA pipeline, which implements the random matrix theory (RMT) approach, to construct the correlation-based relevance network of microbes (Zhou et al., 2010; Deng et al., 2012). A minimum threshold of 0.08 was used for this co-occurrence construction; our inspection of *p*-values for the calculated *r*-values of each built network found them all to be <0.05. The co-occurrence network analysis was then conducted in Gephi v0.9.2 according to Pearson correlation coefficients (*p* < 0.05, |*r*| > 0.8). Relationship maps of key environmental factors and key microbial species were also analyzed in Gephi 0.9.2 (*p* < 0.05, |*r*| > 0.5).

## Results

### Soil physicochemical properties along an elevational gradient of differing slopes

Results revealed that the physicochemical properties of soils along the elevational gradient differed significantly between western and eastern slopes (Table 1). The pH level at each sampling site was acidic, ranging from 4.7 to 6.1, but soil pH was slightly lower on the western than eastern slope, on average. The lowest pH for both slopes occurred at an elevation of 4,100 m. In most sample sites, pH decreased as the elevation increase except for 4,300 m on the eastern slope. TOC and TN in soil showed no significant trends along the elevation gradient of either slope. TOC was similar among elevation sites, ranging from 42 g/kg to 82 g/kg, while the TN varied between 3.2 g/kg and 4.6 g/kg. For both slopes, their highest TOC and C/N values were observed at 4100 m. The content of AK as well as that of CO_3_^2−^ fluctuated with rising elevation, peaking at 3900 m on the western slope. The highest AP content was observed in soil at the lowest elevation on the western slope; AP ranged from 1.9 to 8.2 mg/kg across all sites. There was a significant correlation between pH, TOC, C/N, and CO_3_^2−^ (*p* < 0.05), but these indices were not significantly associated with elevation or slope. The proportion of sand (0.02–2 mm in size), including coarse sand and fine sand particles, was highest in all soil samples, accounting for ~80% of the mechanical fractions (Supplementary Figure S1; Supporting Information).

**Table 1:** Soil properties along altitudinal gradient on different slopes of Mount Segrila.

Sample site	P1	P2	P3	P4	P5	N1	N2	N3	N4	N5
pH	6.09 ± 0.01 f	4.97 ± 0.04 b	5.37 ± 0.04 g	5.21 ± 0.02 f	5.37 ± 0.01 e	4.86 ± 0.05 c	4.70 ± 0.05 a	5.50 ± 0.02 e	5.44 ± 0.04 d	5.22 ± 0.03 e
TN (g kg^−1^)	4.14 ± 0.00 b	3.91 ± 0.01 cd	3.25 ± 0.00 f	4.12 ± 0.01 b	3.60 ± 0.00 e	3.80 ± 0.00 d	4.64 ± 0.01 a	4.01 ± 0.00 c	3.30 ± 0.01 f	3.55 ± 0.00 e
C/N ratio	13.19 ± 0.21 g	18.85 ± 0.49 a	15.86 ± 0.32 d	13.08 ± 0.17 g	16.04 ± 0.06 d	17.66 ± 0.21 c	19.12 ± 0.15 a	13.76 ± 0.33 f	18.36 ± 0.23 b	14.69 ± 0.28 e
AP (mg kg^−1^)	8.09 ± 0.26 b	2.73 ± 0.23 g	7.45 ± 0.14 c	8.20 ± 0.18 b	4.83 ± 0.11 f	1.89 ± 0.27 h	6.76 ± 0.12 d	4.65 ± 0.19 f	5.86 ± 0.08 e	17.20 ± 0.19 a
AK (mg kg^−1^)	197.8 ± 2.7 d	143.7 ± 3.0 h	178.5 ± 5.5 e	166.3 ± 3.8 f	225.7 ± 2.9 c	158.3 ± 4.0 g	285.3 ± 2.0 a	290.7 ± 2.8 a	134.6 ± 1.4 i	257.6 ± 2.0 b
TOC (g kg^−1^)	45.40 ± 0.04 f	67.00 ± 0.13 b	42.40 ± 0.02 g	44.60 ± 0.05 f	46.80 ± 0.04 e	58.60 ± 0.06 c	81.60 ± 0.01 a	47.40 ± 0.03 e	50.60 ± 0.02 d	47.80 ± 0.09 e
CO_3_^2−^ (mg kg^−1^)	13.10 ± 0.07 b	6.20 ± 0.01 ef	7.50 ± 0.01 d	6.10 ± 0.01f	6.60 ± 0.04 e	2.50 ± 0.01 g	2.70 ± 0.00 g	14.00 ± 0.01 a	6.10 ± 0.01 f	8.30 ± 0.02 c

TOC: total organic carbon; TN: total nitrogen; AP: available phosphorus; AK: available potassium; CO_3_^2−^: carbonate content. Number 1, 2, 3, 4, and 5 represent 4,300 m, 4,100 m, 3,900 m, 3,700 m, and 3,500 m, respectively. P and N represent east slope and west slope. The margin of error in the table means standard deviation. Different letters indicate a significant difference between different sites (*P* < 0.05).

### Soil microbial community composition and diversity along the elevational gradient of differing slopes

Illumina sequencing of bacterial 16S rRNA gene amplicons resulted in 465,348 high-quality paired-end reads. After removing the short low-quality reads, singletons, replicates, and chimeras, a total of 3,741 OTUs were obtained from all soil samples based on at least 97% similarity. Sequencing of fungal ITS amplicons led to an initial total of 497,916 paired-end reads; after applying similar data treatments used for bacterial 16S rRNA, a final total of 39,676 sequences remained that clustered into 1,500 OTUs. Together, the bacterial and fungal reads accounted for more than 98% of all obtained soil sequences (Table 2). A rarefaction analysis based on the number of observed species suggested that sequencing depth of bacterial and fungal communities was sufficient for carrying out robust downstream microbial community diversity analyses.

**Table 2:** Diversity indices of bacterial and fungal communities along the lavational gradient on contrasting slopes of Mount Segrila.

Sample site	P1	P2	P3	P4	P5	N1	N2	N3	N4	N5
Bacteria										
Heip	0.229 ± 0.01 b	0.213 ± 0.02 e	0.219 ± 0.02 d	0.153 ± 0.08 j	0.243 ± 0.06 a	0.206 ± 0.02 f	0.187 ± 0.02 h	0.204 ± 0.09 g	0.186 ± 0.02 i	0.226 ± 0.06 c
Shannon	6.056 ± 0.08 a	5.947 ± 0.03 e	5.985 ± 0.04 d	5.409 ± 0.02 j	6.029 ± 0.05 b	5.644 ± 0.06 h	5.491 ± 0.08 i	5.882 ± 0.01 f	5.797 ± 0.05 g	6.025 ± 0.05 c
Chao1	2,178 ± 3.23 c	2,152 ± 4.26 d	2,178 ± 5.12 c	1800 ± 6.35 g	2002 ± 9.46 f	1,694 ± 6.48 h	1,591 ± 7.22 i	2045 ± 3.63 e	2,236 ± 4.85 b	2,358 ± 9.11 a
Coverage	0.984 ± 0.00 c	0.984 ± 0.00 c	0.983 ± 0.00 d	0.986 ± 0.00 b	0.986 ± 0.00 b	0.990 ± 0.00 a	0.990 ± 0.00 a	0.986 ± 0.00 b	0.984 ± 0.00 c	0.983 ± 0.00 d
Fungi										
Heip	0.071 ± 0.04 c	0.052 ± 0.03 d	0.0061 ± 0.04 i	0.036 ± 0.01 g	0.052 ± 0.05 d	0.045 ± 0.07 e	0.037 ± 0.05 f	0.100 ± 0.03 b	0.016 ± 0.05 h	0.108 ± 0.02 a
Shannon	3.152 ± 0.14 c	3.053 ± 0.05 d	0.846 ± 0.23 j	1.985 ± 0.02 g	2.838 ± 0.012 e	2.589 ± 0.23 f	1.904 ± 0.06 h	3.470 ± 0.41 b	1.477 ± 0.03 i	3.883 ± 0.05 a
Chao1	342 ± 8.35 e	403 ± 6.72 c	255 ± 3.12 g	213 ± 1.74 i	368 ± 6.55 d	3,039 ± 8.62 a	189 ± 3.12 j	338 ± 6.23 f	246 ± 4.57 h	485 ± 5.63 b
Coverage	0.999 ± 0.00 a	0.999 ± 0.00 a	0.999 ± 0.00 a	0.999 ± 0.00 a	0.998 ± 0.00 a	0.999 ± 0.00 a	0.999 ± 0.00 a	0.999 ± 0.00 a	0.999 ± 0.00 a	0.998 ± 0.00 b

The numeric suffixes 1, 2, 3, 4, and 5 denote 4,300 m, 4,100 m, 3,900 m, 3,700 m, and 3,500 m, respectively. P and N represent the east-facing slope and west-facing slope, respectively. The margin of error in the table means standard deviation. Different letters indicate a significant difference between different sites (*p* < 0.05).

According to the alpha diversity indices, microbial diversity along elevational gradient of the two slopes was distinct. With respect to the bacterial community, Chao1, Shannon, and heip indices at 3500 m exceeded those of other elevation sites on the western slope. However, soil bacterial community richness, diversity, and evenness at lower elevations, especially 3,700 m, were less than those at higher elevations (i.e., 4,100–4,300 m; Table 2). Whether on the east- or west-facing slope, fungal diversity and richness were greatest at 3500 m, being relatively reduced at middle elevations (i.e., 3,700–4,100 m). PCoA was also used to visualize the dissimilarity matrix of bacterial OTUs among all sampling sites (Supplementary Figure S1; Supporting Information). The first two ordination axes together explained 74% of variability found in bacterial composition. Distinctive β-diversity of microbial communities was generally apparent across the elevation gradient, but those on same slope clustered more, especially for bacteria.

**Figure 2:**
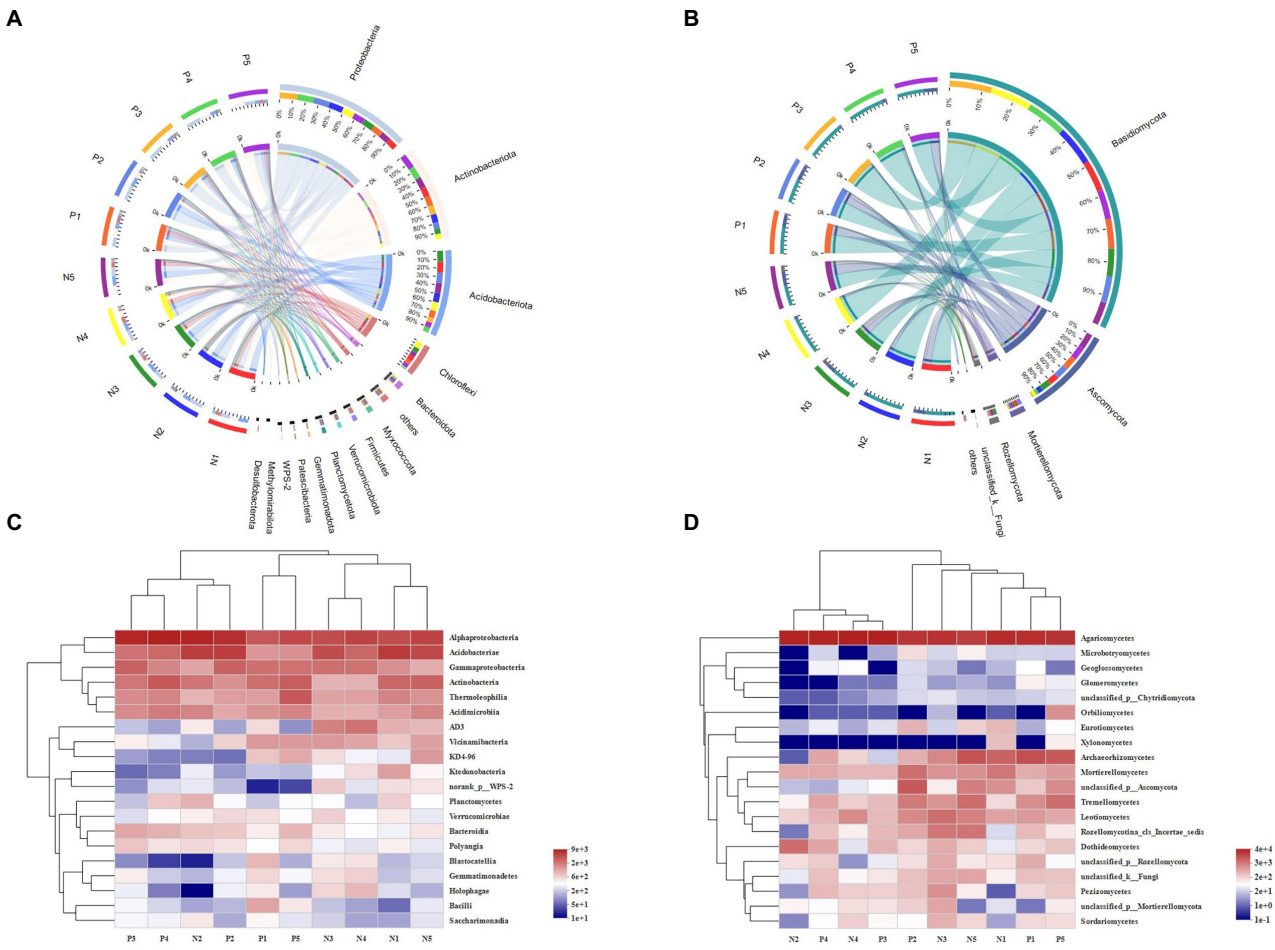
The variation of microbial community composition of soil samples across the elevational gradient on Mount Segrila. Circos of **(A)** bacterial community, and **(B)** fungal community (OTU level). Heatmap comparison analysis of core microbes based on the **(C)** bacterial and **(D)** fungal community composition at class level. P and N represent east slope and west slope, respectively. Different numbers mean different elevations (Number 1, 2, 3, 4, and 5 represent 4,300 m, 4,100 m, 3,900 m, 3,700 m, and 3,500 m, respectively).

In total, we detected 37 bacterial phyla and 13 fungal phyla in all soil samples. As Figure 2 shows, Proteobacteria was the most abundant phylum in soil of both eastern and western slopes. The relative abundance (expressed as proportion) of Proteobacteria, Actinobacteria, Acidobacteria, and Chloroflexi was above 60%, and they were dominant members of bacterial communities in all soil samples. Nevertheless, bacterial composition varied greatly among elevation sites. The relative abundance of Actinobacteria increased significantly at 3500 m and 4,300 m, whereas that of Proteobacteria or Acidobacteria was obviously enhanced at middle elevations. The prevalent fungal phyla were Basidiomycota, Ascomycota, Mortierellamycota, and Rozellomycota. Ascomycota was most abundant at 3500 m and 4,300 m, especially on the western slope. The greatest relative abundance of Mortierellamycota was found in soil at 4100 m on the eastern slope and at 4300 m on the western slope, while Basidiomycota were more abundant at middle elevations (3700–4,100 m).

### Relationship between soil properties, bacterial or fungal community, and their interactions along the elevational gradient of differing slopes

**Figure 3:**
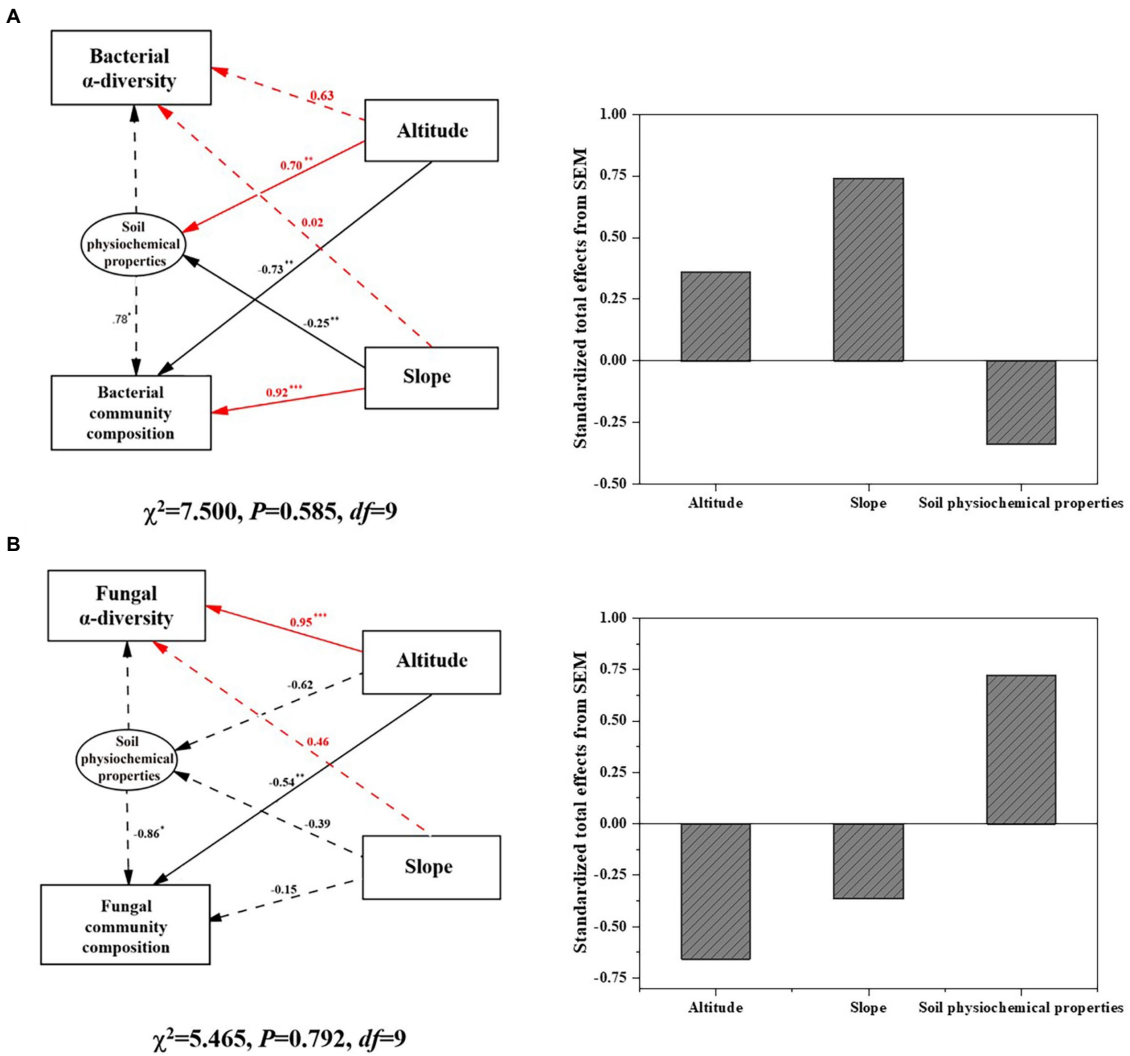
Structural equation model (SEM) illustrating the direct and indirect effects of altitude, slope, and soil physiochemical factors on **(A)** bacterial community, and **(B)** fungal community on Mount Segrila. Arrows depict casual relationships: red lines indicate positive effects, and black lines indicate negative effects. Continuous and dashed arrows indicate significant and nonsignificant relationships, respectively. Significance levels are denoted with *p < 0.1, **p < 0.01, ***p < 0.001. Standardized total effects (direct plus indirect effects) derived from SEM were provided.

SEM as shown in Figure 3 indicated that elevation and slope directly affected bacterial community composition (*p* < 0.01) as well as soil physiochemical properties, which indirectly influenced bacterial composition and diversity. The standardized effect of slope (>70%) on the soil bacterial community was positive and surpassed that of elevation on Mount Segrila. By contrast, elevation had a significantly positive effect on fungal diversity but a negative one upon fungal composition (*p* < 0.01), with no significant correlations between slope and fungal community detected. Standardized total effects of elevation and slope on the fungal community were negative, suggesting different modes by which environmental factors influenced bacterial and fungal communities. In summary, it was found that both elevation and slope had an obvious effect on soil microbial communities but the former exerted a greater influence than the latter upon microbial community composition and diversity, while the bacterial community was more sensitive than fungal community in responding to environmental changes along the elevational gradient of different slopes on Mount Segrila.

**Figure 4:**
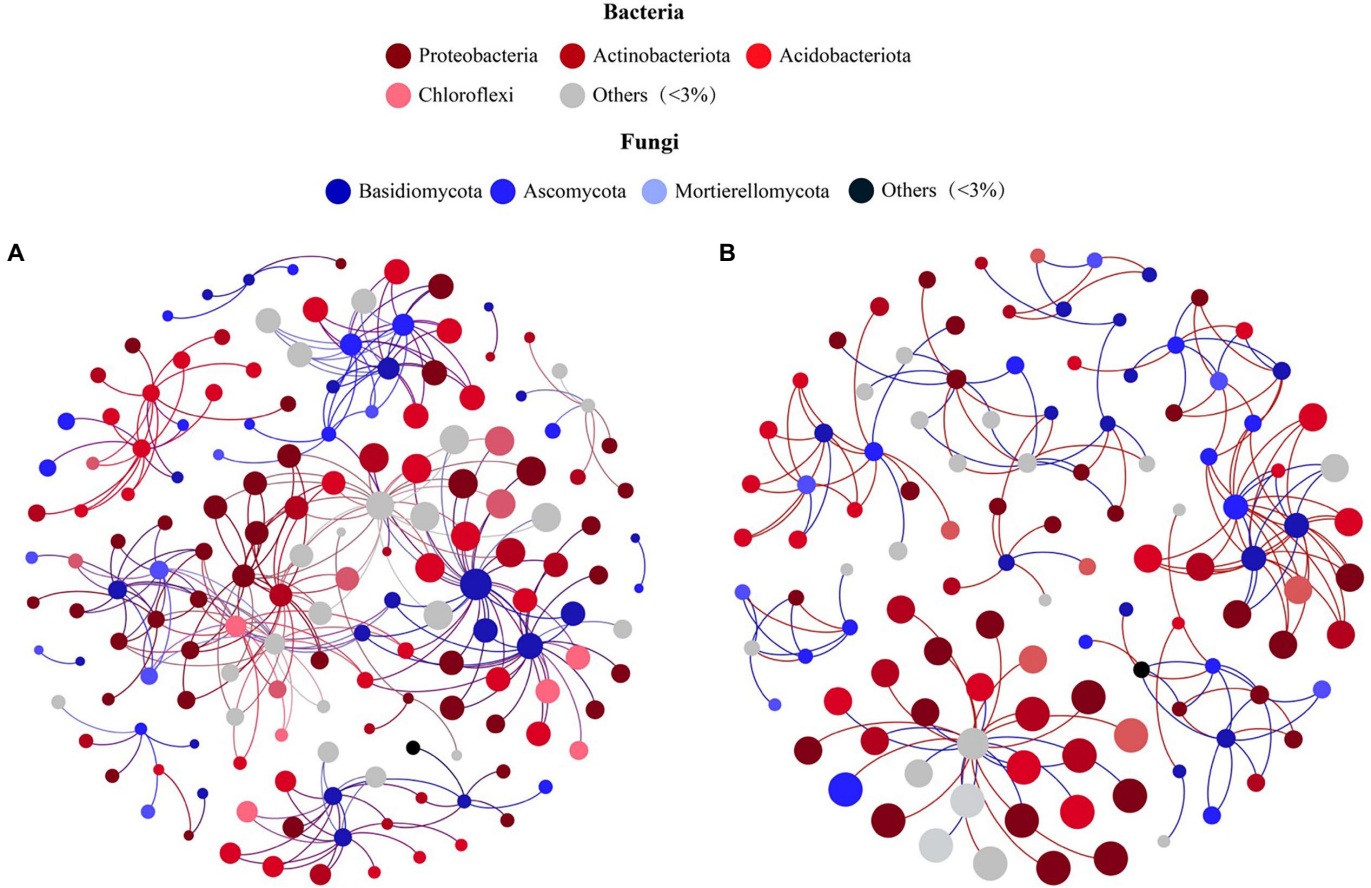
Co-occurrence networks showing the correlation between Bacteria and Fungi of Mount Segrila on **(A)** 3,700, 3,900, 4,100 m and **(B)** 3,500, 4,300 m. Points represent nodes (Species) and lines (edges) represent significant correlations between nodes. Red lines colors represent positive correlations; Blue lines colors represent negative correlations.

According to the PCoA results (Supplementary Figure S1; Supporting Information), bacterial communities of soil samples at 3500 m and 4,300 m clustered together, while those from 3,700, 3,900, and 4,100 m were more similar. According to clustering analysis, two fungal–bacterial co-occurrence networks were constructed to investigate differential biotic interactions. The microbial co-occurrence network of soil taken from middle elevations (3,700 m, 3,900 m, and 4,100 m) incorporated 162 nodes and 276 edges (Figure 4A), while the other network had 115 nodes and 172 edges (Figure 4B), exhibiting a U-shape across the elevational gradient. In these fungal–bacterial co-occurrence networks, bigger nodes had stronger correlations and more edges with other nodes, implying they sustained more complex microbial interactions as key microbes. Comparing the networks among different elevation sites, seven core fungal species were ubiquitous: *Sebacina* sp., *Pleotrichocladium opacum*, *Mortierella humilis*, *Clavaria falcata*, *Clavaria* sp., *Saitozyma* sp., and *Goffeauzyma gastrica* (Supplementary Table S2, S3; Supporting Information), but the key bacteria linked with these fungal species differed among the networks (Supporting Information). This result suggested these hubs, especially core fungi, might be stable and responsive to environmental changes associated with varying elevation and slope orientation as keystone taxa, which may continually reconstruct the fungal–bacterial co-occurrence network based on transmission effects.

**Figure 5:**
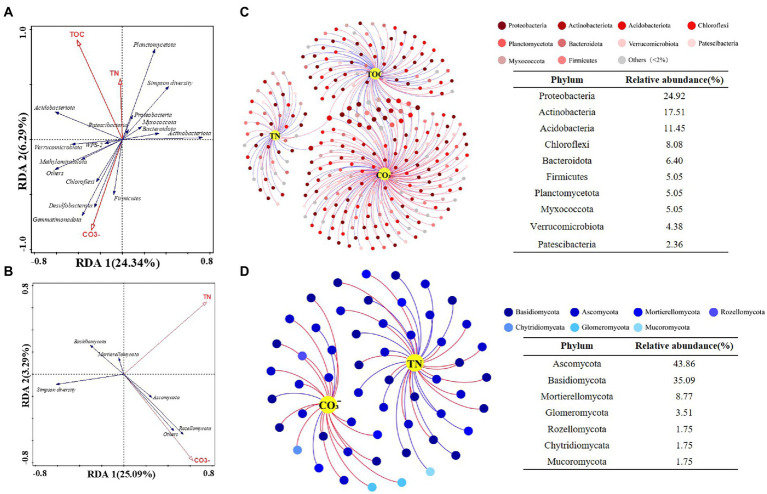
Ordination plots of the results from the redundancy analysis (RDA) to identify the relationships among the **(A)** bacterial and **(B)** fungal taxa (Blue arrows) with the soil physiochemical characteristics (Red arrows). Co-occurrence network visualize the interactions of **(C)** bacterial and **(D)** fungal communities with key environmental factors. Points represent nodes (species) and edges represent significant correlations (*p* < 0.05, |r| < 0.6). Red lines colors represent positive correlations; blue lines colors represent negative correlations.

The RDA let us explore the relationships between soil properties and microbial communities, and to distinguish key environmental factors influencing bacteria and fungi. Monte Carlo tests for all canonical axes were significant (*p* < 0.05), and the first two axes accounted for 30 and 28% of variance in the bacterial community and fungal community, respectively. The forward selection indicated that TOC, CO_3_^2−^, and TN were main drivers of changes to bacterial community composition (*p* < 0.05; Figure 5A), while TN and CO_3_^2−^ contributed significantly to fungal community composition (*p* < 0.05; Figure 5B). Soil nutrient features (e.g., TN, TOC) were significantly correlated with bacterial diversity as well as the relative abundances of Acidobacteriota, Planctomycetota, and Proteobacteria, while those of Desulfobacterota and Firmicutes were strongly correlated with the CO_3_^2−^ content of soil. Concerning the fungi, Ascomycota and Rozellomycota were associated with variation in soil CO_3_^2−^.

Next, the interactions between key soil physiochemical properties and individual species were visualized by building a co-occurrence network. This was based on only strong (|*r*| > 0.6) and significant (*p* < 0.05) correlations; that is, prominent relationships between key environmental factors (TN, TOC and CO_3_^2−^) and bacteria at the species level (Figure 5C) as well as correlations between TN, CO_3_^2−^, and fungal community members (Figure 5D). The bacterial network contained 297 nodes and 320 edges, in which CO_3_^2−^ connected more with bacteria (172 nodes), followed by TOC (97 nodes) and TN (50 nodes). Key bacterial species in the bacterial–environmental network changed significantly vis-à-vis the fungal–bacterial interaction network (Supplementary Table S4; Supporting Information), indicating that soil nutrient features of differing slopes and elevations may influence the bacterial community more and thereby lead to a reorganization of the core fungal–bacterial network. The fungal network consisted of 57 nodes and 55 edges, having more nodes linked to TN than CO_3_^2−^. Intriguingly, one species (*Clavaria* sp.) that had significant correlations with CO_3_^2−^ was also a core species in fungal–bacterial interaction networks (Supplementary Table S4; Supporting Information). Generally, there were more bacteria (> 60%) than fungi in each microbial–environmental network or fungal–bacterial network, suggesting that bacteria contribute more to reorganizing the co-occurrence network along an elevation gradient (3500–4,300 m) on different slopes of Mount Segrila.

## Discussion

Accumulating studies show that the diversity and composition of soil microbial communities varies with elevational distribution characteristics (Xu et al., 2014; Wang et al., 2017; Cui et al., 2019). Climatic conditions can change considerably across different elevations, resulting in differences in vegetation and soil physicochemical properties, which further affects the growth, activity, and composition of soil microbial communities. The climate characterizing high-elevation mountain regions usually confers more environmental stress to affect soil properties, which often has much more of an impact on a soil microbial community than either abiotic or biotic factors such as vegetation (Wang et al., 2020). In this study, soil physicochemical properties as well as microbial communities differed greatly along the sampled elevational gradient on east- vs. west-facing slopes of Mount Segrila, lacking any significant linear relationships (Table 1). SEM results revealed that the elevational gradient significantly shaped both the composition and diversity of bacterial and fungal communities yet slope only affected bacterial community composition (Figure 3). suggesting complexity of environment when considering slope of mountain and variability of bacterial and fungal communities together. Accordingly, this study emphasizes the effects of high elevation on differently oriented slopes upon soil physiochemical properties and microbial community structure, especially the interaction of bacterial and fungal communities.

Soil bacterial community along the elevational gradient of two different slopes in Mount Segrila was significantly affected by TN, TOC, and CO_3_^2−^, while TN and CO_3_^2−^ had distinct effects on fungal community composition. Although different elevations of Mount Segrila can all support alpine forest ecosystem of the Tibetan Plateau, they can differ in the vegetation communities formed there (Li et al., 2014). This could generate disparate nutrient inputs to soil from various litter types of dark coniferous forests versus broadleaved and coniferous mixed forests that may function as microbial habitat filters over a short span of elevation. Meanwhile, local environmental conditions (e.g., light and moisture) can directly affect the activity and growth of vegetation, for example the dynamics of root growth (Huang et al., 2015), which could alter the soil nutrient cycle and influence the microbial community at same elevation due to shady or sunny slopes in the sampling season (summer) of our study. Still, most studies to date have concluded that pH significantly affects bacterial and fungal diversity (Yao et al., 2017; Shen et al., 2020). It is worth noting that CO_3_^2−^ is also considered as another index of soils’ acid–base condition. A reason for why CO_3_^2−^ had an impact upon microbial community instead of pH *per se* could be that CO_3_^2−^ is associated with nutrient ions in soil, confirming nutrients’ content could greatly influence the soil microbial community of Mount Segrila. And carbonate is an important tool for soil ecosystems to cope with climate change due to its essential role in regulating soil pH (Luo et al., 2015), so microorganisms may respond to climate change by adapting to carbonate content in alpine forests with extreme climate variation.

Many studies have reported bacteria and fungi having distinct network patterns in response to differences in environmental factors along elevational gradients but few have concentrated on biotic interactions within soil bacterial and fungal communities (Allison et al., 2010; Yao et al., 2017). For example, Ho et al. (2016) suggested that microbial community composition and nitrogen cycle are closely connected. In our study, a co-occurrence network was constructed to visualize biotic interactions within alpine forest soil along an elevational gradient on Mount Segrila. Interestingly, seven fungal species were present and stable in all sites and had strong correlations with other core microbes, such as *Sebacina* sp., *Pleotrichocladium opacum*, *Mortierella humilis*, to name a few, indicating those species play critical roles in the biotic network of soils on Mount Segrila, also being capable of strong environmental adaptability at 3500–4300 m elevation. Most of them belonged to Ascomycota (42.86%), which were mainly influenced by CO_3_^2−^ and the decomposition of soil organic matter (Figure 5). The *Clavaria* sp. (phylum Ascomycota) was designated a core microbe in Mount Segrila, given its high proportion in the derived fungal–bacterial interaction network, implicating a large influence on organic matter transformation and nitrogen cycling. Core bacteria connected to seven core fungal species in fungal–bacterial interaction network was dissimilar at different elevations, which suggests these bacterial species might serve as a reservoir of genetic and functional diversity and/or buffer ecosystems against species loss or environmental change (Brown et al., 2009). In the bacterial–soil network, we found evidence for some species acting as a “reservoir,” these being correlated with TOC and CO_3_^2−^. The bacteria linked to TOC all belonged to Acidobacteriota or Actinobacteriota, illustrated that these two phyla contributed more to soil organic carbon cycling in Mount Segrila by influencing biotic interactions. Meanwhile, organic carbon compounds in soil had a significant effect upon its bacterial community, contributing chiefly to the soil core microbial interaction network yet not responsible for sustaining the stability of the microbial co-occurrence network.

Although soil microbial community composition and diversity were affected by similar factors (i.e., TN and CO_3_^2−^) driven by the elevational gradient in this study, the responses of bacterial and fungal community were clearly distinct (Figure 3). We found a stronger effect of elevation characteristics on the composition of the bacterial community than that of the fungal community, perhaps due to the greater sensitivity of bacterial communities (Ren et al., 2018). Soil nutrients’ content can be negatively correlated with bacterial diversity, especially heterotrophic bacteria, at higher altitudes (Kumar et al., 2019). Earlier research showed that diazotrophs have selective advantages under low-nutrient environment (Chan, 1994). Most bacteria that are heterotrophic microbes could mineralize organic N compounds and produce various forms of available N for uptake by roots, leading to tight connections between bacteria and forms of soil nutrients (Kuypers et al., 2018). Conversely, N nutrients tend to exert negative effects on fungal community diversity and composition (Cox, 2010; Pellissier et al., 2014; Wang et al., 2015; Siles and Margesin, 2016; Yao et al., 2017) because this is linked with nutrient deposition. Enhanced TN in soil along an elevational gradient due to differing quality litter inputs and nitrogen deposition levels could drive more bacterial growth and diversity given that bacteria are thought to require more N per unit biomass C accumulation in comparison with fungi (Deng et al., 2016).

In this study, we found that Proteobacteria, Actinobacteria, Acidobacteria, and Chloroflexi were dominant in soil, accounting for more than 60% of total bacterial community, similar to survey results reported for the Taibai Mountain and some temperate forests (Ren et al., 2018; Ma et al., 2019; Bayranvand et al., 2021). Microbial community structure at the phylum level at different elevations was distinct but the distribution of bacterial phylum was similar to results from the Changbai Mountain (Heděnec et al., 2019), perhaps because both areas have the same low pH in soil. On Mount Segrila, there was pronounced variation in the relative abundances of Actinobacteria and Acidobacteria along the elevational gradient; this could be due to the slow-growing characteristics, large genomes, and preference for oligotrophic environments of these taxa (Johnston et al., 2019; Lladó et al., 2019). Acidobacteria reportedly prefer a dry and warm environment and has a lower relative abundance under moister and colder soils (Placella et al., 2012; Lauber et al., 2013). Interestingly, Acidobacteria was more common at high altitudes and western slope, unlike Ren et al. (2018) who found the abundance of Acidobacteria was greater at low altitudes in warm and low-humidity environments. In our study, the Actinobacteria was more abundant at lowest elevations and relatively less so at higher elevations, likely because Actinobacteria spp. are oligotrophic and common in environments with limited soil nutrients, such those typically found at high elevations in mountains regions (Yao et al., 2017; Johnston et al., 2019).

Compared with its bacterial community, the fungal community of Mount Segrila’s soils was more stably distributed along the elevational gradient, with Basidiomycota and Ascomycota comprising more than 80% of sampled fungi. Many researchers believe the distribution of fungal community composition is influenced principally by climatic conditions and soil physical properties (e.g., Vetrovsky et al., 2019). For example, fungal communities changed substantially under experimental warming conditions, indicating that climate change could significantly impact soil fungi (Allison et al., 2010; Ihsan et al., 2021). In the present study, however, the abundance of those two phyla did not show a clear geographical signature, for which a plausible explanation could be the dense needle-leaf litter in alpine forest of Mount Segrila. Furthermore, it is widely reported that the main ecosystem role of Basidiomycota and Ascomycota is to decompose organic matter as well as inter-root sediments, a process influenced by organic carbon content of soil (Hannula et al., 2012; Clemmensen et al., 2013). Because of the low temperature and slow decomposition of litter, the surface of the soil in Mount Segrila is covered with a thick layer of litter after years of accumulation. As fungi’s main function is to decompose organic C compounds occurring in recalcitrant forms (Treseder et al., 2016) and it can be stable in extreme climates, so fungi such as Basidiomycota and Ascomycota play an important role at high elevations.

## Conclusion

The results here demonstrated that soil physiochemical characteristics and microbial communities vary considerably along an elevational gradient on Mount Segrila. The Proteobacteria, Actinobacteria, Acidobacteria, and Chloroflexi all occur in high proportions in the soil bacterial community, while the Basidiomycota and Ascomycota are major phyla in the soil fungal community. Fungal diversity and composition are both correlated with TN and CO_3_^2−^, while the bacterial community is jointly influenced by TN, TOC, and CO_3_^2−^. Elevation rather than slope orientation is the main driver of soil microbial community properties of Mount Segrila. Biotic interactions between core bacteria and fungi as affected by edaphic factors could be deconstructed and reorganized along the elevational gradient. Fungi, especially Ascomycota, are important for shaping and sustaining the interrelationship of bacterial and fungal co-occurrence networks. Fungi are evidently stable, but bacteria contribute more to reorganizing the soil microbial network across the elevation span of 3,500–4,300 m on Mount Segrila.

## Data availability statement

The original contributions presented in the study are publicly available. This data can be found at: NCBI Sequence Read Archive (SRA) database (Accession Number: SRP331508).

## Author contributions

TM: methodology, data curation, conceptualization, and funding acquisition. XZ: data curation, visualization, and writing—original draft. RW: conceptualization, formal analysis, validation, and writing—review and editing. RL: methodology and visualization. XS: validation and funding acquisition. JL: conceptualization and resources. YW: conceptualization, funding acquisition, resources, and project administration. All authors contributed to the article and approved the submitted version.

## Funding

This work was financially supported by the Open Research Fund from the Key Laboratory of Forest Ecology in Tibet Plateau (Tibet Agriculture and Animal Husbandry University), Ministry of Education, China [grant XZAJYBSYS-2020-02], National Natural Science Foundation of China (42007031 and 31960013), Joint project of China Agricultural University and Tibet Agricultural & Animal Husbandry University, and Central government guides local projects (XZ202101YD0013C), The Independent Research Project of Science and Technology Innovation Base in Tibet Autonomous (XZ2022JR0007G).

## Conflict of interest

The authors declare that the research was conducted in the absence of any commercial or financial relationships that could be construed as a potential conflict of interest.

## Publisher’s note

All claims expressed in this article are solely those of the authors and do not necessarily represent those of their affiliated organizations, or those of the publisher, the editors and the reviewers. Any product that may be evaluated in this article, or claim that may be made by its manufacturer, is not guaranteed or endorsed by the publisher.
