# Supplementary material for: Linkages and key factors between soil bacterial and fungal communities along an altitudinal gradient of different slopes on mount Segrila, Tibet, China
Source: Front Microbiol. 2022 Oct 28;13:1024198. doi: 10.3389/fmicb.2022.1024198 (PMC9649828; doi:10.3389/fmicb.2022.1024198)
Supplement: Supplementary file 1 [file Data_Sheet_1.docx]

**Supplementary Materials**

**Linkages and key factors between soil bacterial and fungal communities along an altitudinal gradient on Mount Segrila, Tibet, China**

Tiantian Ma^1^, Xinjun Zhang^2^, Ruihong Wang^2^, Rui Liu^1^, Xiaoming Shao^1^, Ji Li^1,3^, Yuquan Wei^1,3,*^

*^1^ College of Resources and Environmental Science, Beijing Key Laboratory of Biodiversity and Organic Farming, China Agricultural University, 100193, Beijing, China*

*^2^ Institute of Tibet Plateau Ecology, Tibet Agricultural & Animal Husbandry University, and Key Laboratory of Forest Ecology in Tibet Plateau (Tibet Agricultural & Animal Husbandry University), Ministry of Education, Nyingchi, Tibet 860000, China*

*^3^ Organic Recycling Institute (Suzhou) of China Agricultural University, Wuzhong District, Suzhou 215128, China*

^*^ Corresponding author

E-mail addresses: weiyq2013@gmail.com (Yuquan Wei)

Supporting Information Includes:

- 4 tables

- 1 figure

**Table S1.** Soil physical properties of mechanical composition at different sites from varying altitudes on Mount Segrila.

|  | **Clay** | **Slit** | **Fine sand** | **Coarse sand** | **Sand** |
| --- | --- | --- | --- | --- | --- |
| **N1** | 15.55% | 16.84% | 41.61% | 25.79% | 67.61% |
| **N2** | 15.19% | 20.63% | 48.18% | 15.79% | 64.18% |
| **N3** | 12.39% | 27.52% | 42.09% | 17.77% | 60.09% |
| **N4** | 18.93% | 21.44% | 31.63% | 28.28% | 59.63% |
| **N5** | 12.38% | 23.91% | 46.71% | 16.92% | 63.71% |
| **P1** | 8.58% | 25.75% | 38.67% | 27.42% | 65.67% |
| **P2** | 13.86% | 23.30% | 57.84% | 4.55% | 62.84% |
| **P3** | 11.37% | 17.56% | 27.07% | 44.39% | 71.07% |
| **P4** | 12.73% | 21.21% | 49.06% | 15.54% | 65.06% |
| **P5** | 15.87% | 22.13% | 50.00% | 12.11% | 62.00% |

P and N represent east slope and west slope, respectively. Different numbers mean different elevations (Number 1, 2, 3, 4, and 5 represent 4300 m, 4100 m, 3900 m, 3700 m, and 3500 m, respectively).


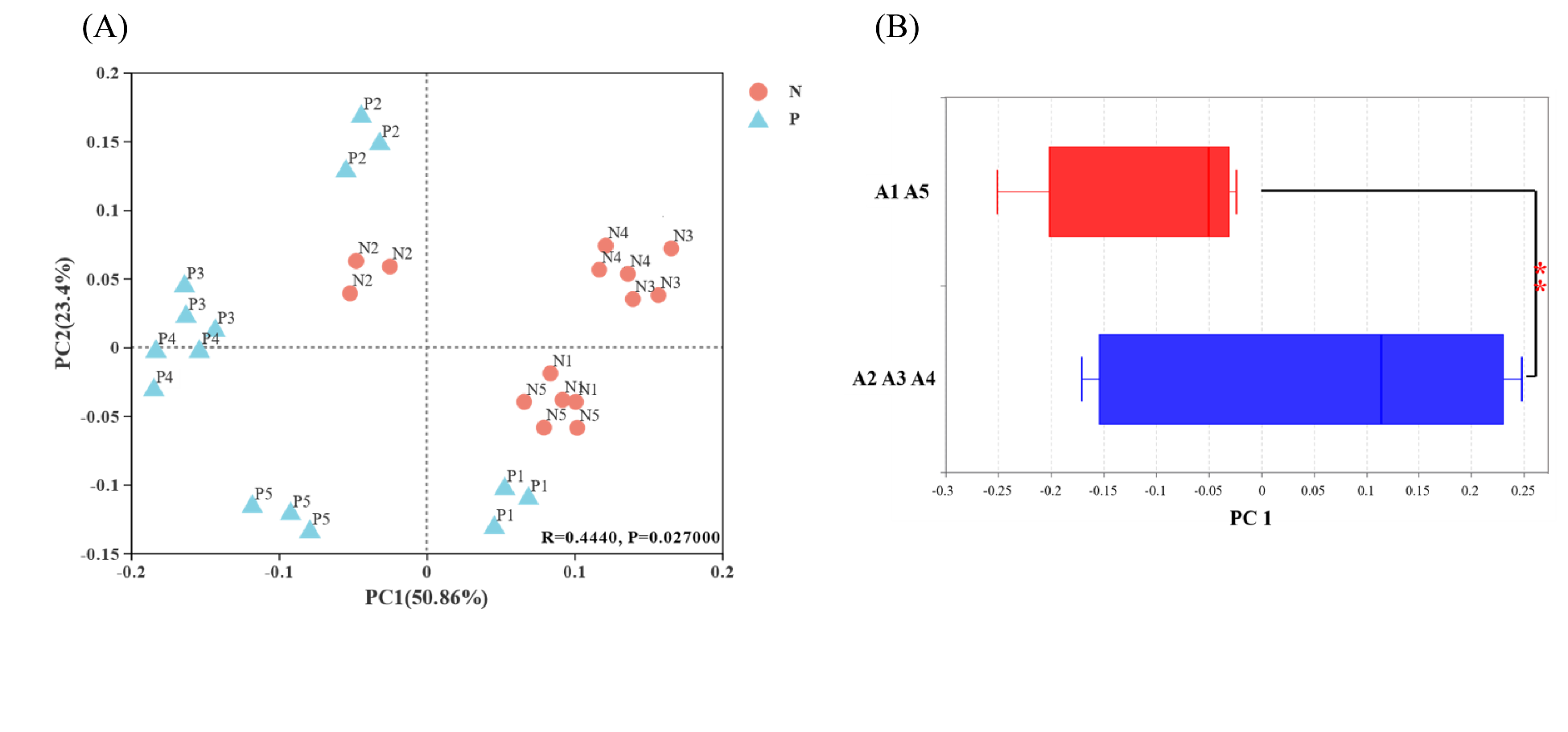


**Figure S1.** The variation of microbial community composition of soil samples across the elevational gradient on Mount Segrila. Principal co-ordinates analysis (PCoA) based on the bacterial (A) and signification difference comparison between different group of (B) bacterial community (OTU level). P and N represent east slope and west slope, respectively. A represents altitudes. Different numbers mean different elevations (Number 1, 2, 3, 4, and 5 represent 4300 m, 4100 m, 3900 m, 3700 m, and 3500 m, respectively).

| **Table S2(a). Neighbors of core microbes in fungal-bacterial co-occurrence network in 3700, 3900, 4100 m** |
| --- |

| **Key Species** | **Neighbors** |
| --- | --- |

| ***Clavaria* sp** |  |
| --- | --- |
|  | *unclassified_o__Agaricales* |
|  | *Pleotrichocladium_opacum* |
|  | *unclassified_o__Helotiales* |
|  | *Sebacina_sp* |
|  | *Archaeorhizomycetes_sp* |
|  | *Goffeauzyma_gastrica* |
|  | *Pezoloma_ericae* |
|  | *unclassified_g__norank_f__Xanthobacteraceae* |
|  | *uncultured_bacterium_g__norank_f__norank_o__Acidobacteriales* |
|  | *unclassified_g__Candidatus_Solibacter* |
|  | *uncultured_bacterium_g__Pseudonocardia* |
|  | *unclassified_f__Comamonadaceae* |
|  | *uncultured_bacterium_g__norank_f__norank_o__norank_c__AD3* |
|  | *unclassified_g__norank_f__Micropepsaceae* |
|  | *metagenome_g__Gaiella* |
|  | *unclassified_c__Alphaproteobacteria* |
|  | *uncultured_bacterium_g__Granulicella* |
|  | *uncultured_bacterium_g__norank_f__norank_o__norank_c__norank_p__WPS-2* |
|  | *uncultured_bacterium_g__norank_f__norank_o__Subgroup_2* |
|  | *unclassified_g__norank_f__norank_o__Elsterales* |
|  | *uncultured_Acidobacteriaceae_bacterium_g__norank_f__norank* |
|  | *uncultured_bacterium_g__norank_f__A21b* |
|  | *unclassified_g__norank_f__Amb-16S-1323* |
|  | *unclassified_g__Bacillus* |
|  | *unclassified_g__Bryobacter* |
|  | *unclassified_g__HSB_OF53-F07* |
| ***Clavaria falcata*** |  |
|  | *Mortierella_horticola* |
|  | *Pleotrichocladium_opacum* |
|  | *Amphinema_sp* |
|  | *Sebacina_sp* |
|  | *Goffeauzyma_gastrica* |
|  | *Tomentella_stuposa* |
|  | *unclassified_g__norank_f__Xanthobacteraceae* |
|  | *uncultured_Acidimicrobiales_bacterium_g__norank_f__norank* |
|  | *unclassified_g__Gemmatimonas* |
|  | *unclassified_f__Comamonadaceae* |
|  | *unclassified_g__norank_f__Vicinamibacteraceae* |
|  | *unclassified_g__norank_f__Micropepsaceae* |
|  | *uncultured_bacterium_g__norank_f__norank_o__norank_c__KD4-96* |
|  | *uncultured_bacterium_g__Galbitalea* |
|  | *metagenome_g__Gaiella* |
|  | *unclassified_c__Alphaproteobacteria* |
|  | *unclassified_c__Actinobacteria* |
|  | *unclassified_g__norank_f__Acetobacteraceae* |
|  | *unclassified_o__Frankiales* |
|  | *uncultured_bacterium_g__norank_f__norank_o__Vicinamibacterales* |
|  | *unclassified_g__norank_f__norank_o__Elsterales* |
|  | *uncultured_Bellilinea_sp._g__norank_f__Anaerolineaceae* |
|  | *uncultured_bacterium_g__norank_f__A21b* |
|  | *unclassified_g__norank_f__Amb-16S-1323* |
|  | *unclassified_g__Nakamurella* |
|  | *unclassified_g__Bryobacter* |
|  | *unclassified_g__HSB_OF53-F07* |
|  | *metagenome_g__norank_f__67-14* |
|  | *Nocardioides_sp._g__Nocardioides* |
| ***Saitozyma* sp** |  |
|  | *Mortierella_humilis* |
|  | *Russula_cyanoxantha* |
|  | *Russula_puellaris* |
|  | *uncultured_bacterium_g__norank_f__norank_o__Elsterales* |
|  | *unclassified_g__mle1-7* |
|  | *unclassified_o__Burkholderiales* |
|  | *uncultured_bacterium_g__norank_f__norank_o__norank_c__MB-A2-108* |
|  | *unclassified_g__norank_f__norank_o__Subgroup_17* |
|  | *unclassified_g__norank_f__norank_o__norank_c__Acidimicrobiia* |
|  | *unclassified_g__Acidothermus* |
|  | *metagenome_g__Reyranella* |
|  | *metagenome_g__norank_f__Micropepsaceae* |
|  | *unclassified_g__Candidatus_Udaeobacter* |
|  | *uncultured_Acidobacteria_bacterium_g__Acidipila* |
|  | *uncultured_bacterium_g__norank_f__norank_o__Rokubacteriales* |
|  | *uncultured_Chloroflexi_bacterium_g__norank_f__norank_o__norank_c__KD4-96* |
|  | *unclassified_o__Saccharimonadales* |
|  | *uncultured_bacterium_g__IS-44* |
|  | *uncultured_bacterium_g__norank_f__norank_o__Subgroup_7* |
|  | *uncultured_bacterium_g__Geobacter* |
|  | *unclassified_g__Pedomicrobium* |
|  | *unclassified_g__Occallatibacter* |
| ***Mortierella humilis*** |  |
|  | *unclassified_c__Eurotiomycetes* |
|  | *metagenome_g__norank_f__norank_o__Gaiellales* |
|  | *uncultured_forest_soil_bacterium_g__norank_f__norank_o__Subgroup_2* |
|  | *uncultured_bacterium_g__norank_f__norank_o__Elsterales* |
|  | *unclassified_g__norank_f__norank_o__norank_c__KD4-96* |
|  | *uncultured_actinobacterium_g__norank_f__norank_o__norank_c__MB-A2-108* |
|  | *uncultured_bacterium_g__norank_f__norank_o__norank_c__MB-A2-108* |
|  | *unclassified_g__norank_f__norank_o__Subgroup_17* |
|  | *uncultured_bacterium_g__Roseiarcus* |
|  | *unclassified_g__Arthrobacter* |
|  | *unclassified_g__norank_f__norank_o__norank_c__Acidimicrobiia* |
|  | *unclassified_g__Acidothermus* |
|  | *metagenome_g__Reyranella* |
|  | *unclassified_g__Candidatus_Udaeobacter* |
|  | *uncultured_forest_soil_bacterium_g__norank_f__norank_o__Acidobacteriales* |
|  | *unclassified_g__Candidatus_Xiphinematobacter* |
|  | *uncultured_Acidobacteria_bacterium_g__Acidipila* |
|  | *unclassified_g__norank_f__Gemmataceae* |
|  | *uncultured_forest_soil_bacterium_g__norank_f__norank_o__norank_c__Acidimicrobiia* |
|  | *uncultured_Acidobacteria_bacterium_g__Occallatibacter* |
|  | *uncultured_bacterium_g__norank_f__norank_o__Rokubacteriales* |
|  | *unclassified_g__Hyphomicrobium* |
|  | *metagenome_g__norank_f__TRA3-20* |
|  | *uncultured_bacterium_g__Oryzihumus* |
|  | *uncultured_bacterium_g__IS-44* |
|  | *unclassified_g__Conexibacter* |
|  | *unclassified_g__norank_f__norank_o__Vicinamibacterales* |
|  | *uncultured_bacterium_g__norank_f__norank_o__Subgroup_7* |
|  | *uncultured_Gemmatimonadales_bacterium_g__norank_f__Gemmatimonadaceae* |
|  | *metagenome_g__norank_f__Acetobacteraceae* |
|  | *uncultured_bacterium_g__Geobacter* |
|  | *metagenome_g__norank_f__Methyloligellaceae* |
|  | *unclassified_g__Pedomicrobium* |
|  | *uncultured_bacterium_g__Acidothermus* |
| ***Goffeauzyma gastrica*** |  |
|  | *Tomentella_stuposa* |
|  | *unclassified_g__norank_f__Xanthobacteraceae* |
|  | *uncultured_Acidimicrobiales_bacterium_g__norank_f__norank* |
|  | *unclassified_g__norank_f__norank_o__Subgroup_7* |
|  | *uncultured_bacterium_g__norank_f__norank_o__Acidobacteriales* |
|  | *unclassified_g__Candidatus_Solibacter* |
|  | *uncultured_bacterium_g__Pseudonocardia* |
|  | *unclassified_g__Gemmatimonas* |
|  | *Clostridium_estertheticum* |
|  | *uncultured_bacterium_g__norank_f__norank_o__norank_c__AD3* |
|  | *unclassified_g__norank_f__Micropepsaceae* |
|  | *unclassified_g__Mycobacterium* |
|  | *unclassified_c__Alphaproteobacteria* |
|  | *unclassified_g__Iamia* |
|  | *unclassified_g__norank_f__Acetobacteraceae* |
|  | *uncultured_bacterium_g__norank_f__norank_o__Subgroup_2* |
|  | *uncultured_Acidobacteriaceae_bacterium_g__norank_f__norank* |
|  | *uncultured_bacterium_g__norank_f__A21b* |
|  | *unclassified_g__norank_f__Amb-16S-1323* |
|  | *unclassified_g__Nakamurella* |
|  | *unclassified_g__RB41* |
|  | *metagenome_g__norank_f__67-14* |
|  | *Nocardioides_sp._g__Nocardioides* |
|  | *Pleotrichocladium_opacum* |
| ***Sebacina* sp** |  |
|  | *Archaeorhizomycetes_sp* |
|  | *unclassified_f__Mortierellaceae* |
|  | *uncultured_bacterium_g__norank_f__norank_o__Gaiellales* |
|  | *uncultured_Acidimicrobiales_bacterium_g__norank_f__norank* |
|  | *uncultured_Chloroflexi_bacterium_g__norank_f__norank_o__norank_c__AD3* |
|  | *uncultured_bacterium_g__norank_f__norank_o__Acidobacteriales* |
|  | *unclassified_g__Candidatus_Solibacter* |
|  | *uncultured_bacterium_g__Pseudonocardia* |
|  | *unclassified_g__Gemmatimonas* |
|  | *unclassified_f__Comamonadaceae* |
|  | *uncultured_bacterium_g__norank_f__norank_o__norank_c__AD3* |
|  | *metagenome_g__Gaiella* |
|  | *unclassified_g__norank_f__Acetobacteraceae* |
|  | *uncultured_Acidobacteria_bacterium_g__norank_f__norank_o__Subgroup_2* |
|  | *uncultured_bacterium_g__norank_f__norank_o__Subgroup_2* |
|  | *uncultured_bacterium_g__norank_f__norank_o__JG36-TzT-191* |
|  | *unclassified_g__norank_f__norank_o__Elsterales* |
|  | *uncultured_Acidobacteriaceae_bacterium_g__norank_f__norank* |
|  | *unclassified_g__norank_f__SC-I-84* |
|  | *unclassified_g__Nakamurella* |
|  | *uncultured_Acidobacteria_bacterium_g__Bryobacter* |
|  | *uncultured_bacterium_g__Aquisphaera* |
|  | *unclassified_g__Bryobacter* |
|  | *unclassified_g__HSB_OF53-F07* |
|  | *metagenome_g__norank_f__67-14* |
|  | *Nocardioides_sp._g__Nocardioides* |
|  | *Sebacina_sp* |
| ***Pleotrichocladium opacum*** |  |
|  | *Archaeorhizomycetes_sp* |
|  | *unclassified_f__Mortierellaceae* |
|  | *uncultured_bacterium_g__norank_f__norank_o__Gaiellales* |
|  | *uncultured_Acidimicrobiales_bacterium_g__norank_f__norank* |
|  | *uncultured_Chloroflexi_bacterium_g__norank_f__norank_o__norank_c__AD3* |
|  | *uncultured_bacterium_g__norank_f__norank_o__Acidobacteriales* |
|  | *unclassified_g__Candidatus_Solibacter* |
|  | *uncultured_bacterium_g__Pseudonocardia* |
|  | *unclassified_g__Gemmatimonas* |
|  | *unclassified_f__Comamonadaceae* |
|  | *uncultured_bacterium_g__norank_f__norank_o__norank_c__AD3* |
|  | *metagenome_g__Gaiella* |
|  | *unclassified_g__norank_f__Acetobacteraceae* |
|  | *uncultured_Acidobacteria_bacterium_g__norank_f__norank_o__Subgroup_2* |
|  | *uncultured_bacterium_g__norank_f__norank_o__Subgroup_2* |
|  | *uncultured_bacterium_g__norank_f__norank_o__JG36-TzT-191* |
|  | *unclassified_g__norank_f__norank_o__Elsterales* |
|  | *uncultured_Acidobacteriaceae_bacterium_g__norank_f__norank* |
|  | *unclassified_g__norank_f__SC-I-84* |
|  | *unclassified_g__Nakamurella* |
|  | *uncultured_Acidobacteria_bacterium_g__Bryobacter* |
|  | *uncultured_bacterium_g__Aquisphaera* |
|  | *unclassified_g__Bryobacter* |
|  | *unclassified_g__HSB_OF53-F07* |
|  | *metagenome_g__norank_f__67-14* |

**Table S2(b). Neighbors of core microbes in fungal-bacterial co-occurrence network in 3500, 4300 m**

| **Key Species** | **Neighbors** |
| --- | --- |
| ***Clavaria* sp** | *uncultured_bacterium_g__norank_f__norank_o__Acidobacteriales* |
|  | *unclassified_g__Candidatus_Solibacter* |
|  | *uncultured_bacterium_g__Pseudonocardia* |
|  | *uncultured_bacterium_g__norank_f__norank_o__norank_c__AD3* |
|  | *unclassified_o__Acidobacteriales* |
|  | *uncultured_bacterium_g__norank_f__norank_o__Subgroup_2* |
|  | *uncultured_Acidobacteriaceae_bacterium_g__norank_f__norank* |
|  |  |
| ***Clavaria falcata*** |  |
|  | *unclassified_f__Micromonosporaceae* |
|  | *bacterium_Ellin6089* |
|  | *unclassified_g__Gemmatimonas* |
|  | *unclassified_g__norank_f__Acetobacteraceae* |
|  | *uncultured_bacterium_g__norank_f__Ilumatobacteraceae* |
|  | *unclassified_g__Nakamurella* |
|  | *metagenome_g__norank_f__67-14* |
|  | *Nocardioides_sp._g__Nocardioides* |
| ***Saitozyma* sp** |  |
|  | *uncultured_bacterium_g__norank_f__norank_o__Elsterales* |
|  | *unclassified_g__norank_f__A0839* |
|  | *uncultured_bacterium_g__norank_f__norank_o__norank_c__MB-A2-108* |
|  | *uncultured_bacterium_g__norank_f__norank_o__Rokubacteriales* |
|  | *uncultured_bacterium_g__norank_f__norank_o__Subgroup_7* |
|  | *unclassified_g__GOUTA6* |
|  | *uncultured_bacterium_g__Geobacter* |
|  | *unclassified_g__Pedomicrobium* |
| ***Goffeauzyma gastrica*** |  |
|  | *unclassified_g__norank_f__Xanthobacteraceae* |
|  | *uncultured_gamma_proteobacterium_g__Acidibacter* |
|  | *unclassified_g__norank_f__Micropepsaceae* |
|  | *unclassified_c__Alphaproteobacteria* |
|  | *uncultured_bacterium_g__norank_f__A21b* |
|  | *unclassified_g__norank_f__Amb-16S-1323* |
| ***Pleotrichocladium opacum*** | |
|  | *unclassified_f__Comamonadaceae* |
|  | *metagenome_g__Gaiella* |
|  | *unclassified_g__norank_f__norank_o__Elsterales* |
|  | *uncultured_bacterium_g__Luedemannella* |
|  | *unclassified_g__Bryobacter* |
|  | *unclassified_g__HSB_OF53-F07* |
|  | *Ilyonectria_rufa* |
|  | *Sebacina_sp* |
| ***Mortierella humilis*** |  |
|  | *unclassified_g__norank_f__Gemmatimonadaceae* |
|  | *unclassified_g__norank_f__norank_o__Subgroup_17* |
|  | *unclassified_g__norank_f__norank_o__norank_c__Acidimicrobiia* |
|  | *unclassified_g__Acidothermus* |
|  | *metagenome_g__Reyranella* |
|  | *unclassified_g__Candidatus_Udaeobacter* |
|  | *uncultured_planctomycete_g__norank_f__Gemmataceae* |
|  | *uncultured_Acidobacteria_bacterium_g__Acidipila* |
|  | *uncultured_bacterium_g__IS-44* |
|  | *uncultured_beta_proteobacterium_g__norank_f__SC-I-84* |
| ***Sebacina* sp** |  |
|  | *unclassified_f__Comamonadaceae* |
|  | *metagenome_g__Gaiella* |
|  | *unclassified_g__norank_f__norank_o__Elsterales* |
|  | *uncultured_bacterium_g__Luedemannella* |
|  | *unclassified_g__Bryobacter* |
|  | *unclassified_g__HSB_OF53-F07* |
|  | *Ilyonectria_rufa* |
|  | *Pleotrichocladium_opacum* |

| **Species**  **Table S3. Information of different speices connect to core microbes in different altitudes** | **Phylum** | **Class** | **Order** | **Family** | **Genus** |
| --- | --- | --- | --- | --- | --- |
| **3700, 3900,4100 m** | | | | | |
| *unclassified_o__Agaricales* | Basidiomycota | Agaricomycetes | Agaricales | unclassified_o__Agaricales | *unclassified_o__Agaricales* |
| *unclassified_o__Helotiales* | Ascomycota | Leotiomycetes | Helotiales | unclassified_o__Helotiales | *unclassified_o__Helotiales* |
| *Archaeorhizomycetes_sp* | Ascomycota | Archaeorhizomycetes | unclassified_c__Archaeorhizomycetes | unclassified_c__Archaeorhizomycetes | *unclassified_c__Archaeorhizomycetes* |
| *Pezoloma_ericae* | Ascomycota | Leotiomycetes | Helotiales | Leotiaceae | *Pezoloma* |
| *uncultured_bacterium_g__Granulicella* | Acidobacteriota | Acidobacteriae | Acidobacteriales | Acidobacteriaceae_Subgroup_1 | *Granulicella* |
| *uncultured_bacterium_g__norank_f__norank_o__norank_c__norank_p__WPS-2* | WPS-2 | norank_p__WPS-2 | norank_c__norank_p__WPS-2 | norank_o__norank_c__norank_p__WPS-2 | *norank_f__norank_o__norank_c__norank_p__WPS-2* |
| *unclassified_g__Bacillus* | Firmicutes | Bacilli | Bacillales | Bacillaceae | *Bacillus* |
| *Mortierella_horticola* | Mortierellomycota | Mortierellomycetes | Mortierellales | Mortierellaceae | *Mortierella* |
| *Amphinema_sp* | Basidiomycota | Agaricomycetes | Atheliales | Atheliaceae | *Amphinema* |
| *Tomentella_stuposa* | Basidiomycota | Agaricomycetes | Thelephorales | Thelephoraceae | *Tomentella* |
| *uncultured_Acidimicrobiales_bacterium_g__norank_f__norank* | Actinobacteriota | Acidimicrobiia | IMCC26256 | norank_o__IMCC26256 | *norank_f__norank_o__IMCC26256* |
| *unclassified_g__norank_f__Vicinamibacteraceae* | Acidobacteriota | Vicinamibacteria | Vicinamibacterales | Vicinamibacteraceae | *norank_f__Vicinamibacteraceae* |
| *uncultured_bacterium_g__norank_f__norank_o__norank_c__KD4-96* | Chloroflexi | KD4-96 | norank_c__KD4-96 | norank_o__norank_c__KD4-96 | *norank_f__norank_o__norank_c__KD4-96* |
| *uncultured_bacterium_g__Galbitalea* | Actinobacteriota | Actinobacteria | Micrococcales | Microbacteriaceae | *Galbitalea* |
| *unclassified_c__Actinobacteria* | Actinobacteriota | Actinobacteria | unclassified_c__Actinobacteria | unclassified_c__Actinobacteria | *unclassified_c__Actinobacteria* |
| *unclassified_o__Frankiales* | Actinobacteriota | Actinobacteria | Frankiales | unclassified_o__Frankiales | *unclassified_o__Frankiales* |
| *uncultured_bacterium_g__norank_f__norank_o__Vicinamibacterales* | Acidobacteriota | Vicinamibacteria | Vicinamibacterales | norank_o__Vicinamibacterales | *norank_f__norank_o__Vicinamibacterales* |
| *uncultured_Bellilinea_sp._g__norank_f__Anaerolineaceae* | Chloroflexi | Anaerolineae | Anaerolineales | Anaerolineaceae | *norank_f__Anaerolineaceae* |
| *Russula_cyanoxantha* | Basidiomycota | Agaricomycetes | Russulales | Russulaceae | *Russula* |
| *Russula_puellaris* | Basidiomycota | Agaricomycetes | Russulales | Russulaceae | *Russula* |
| *unclassified_g__mle1-7* | Proteobacteria | Gammaproteobacteria | Burkholderiales | Nitrosomonadaceae | *mle1-7* |
| *unclassified_o__Burkholderiales* | Proteobacteria | Gammaproteobacteria | Burkholderiales | unclassified_o__Burkholderiales | *unclassified_o__Burkholderiales* |
| *metagenome_g__norank_f__Micropepsaceae* | Proteobacteria | Alphaproteobacteria | Micropepsales | Micropepsaceae | *norank_f__Micropepsaceae* |
| *uncultured_Chloroflexi_bacterium_g__norank_f__norank_o__norank_c__KD4-96* | Chloroflexi | KD4-96 | norank_c__KD4-96 | norank_o__norank_c__KD4-96 | *norank_f__norank_o__norank_c__KD4-96* |
| *unclassified_o__Saccharimonadales* | Patescibacteria | Saccharimonadia | Saccharimonadales | unclassified_o__Saccharimonadales | *unclassified_o__Saccharimonadales* |
| *unclassified_g__Occallatibacter* | Acidobacteriota | Acidobacteriae | Acidobacteriales | Acidobacteriaceae_Subgroup_1 | *Occallatibacter* |
| *unclassified_c__Eurotiomycetes* | Ascomycota | Eurotiomycetes | unclassified_c__Eurotiomycetes | unclassified_c__Eurotiomycetes | *unclassified_c__Eurotiomycetes* |
| *metagenome_g__norank_f__norank_o__Gaiellales* | Actinobacteriota | Thermoleophilia | Gaiellales | norank_o__Gaiellales | *norank_f__norank_o__Gaiellales* |
| *uncultured_forest_soil_bacterium_g__norank_f__norank_o__Subgroup_2* | Acidobacteriota | Acidobacteriae | Subgroup_2 | norank_o__Subgroup_2 | *norank_f__norank_o__Subgroup_2* |
| *unclassified_g__norank_f__norank_o__norank_c__KD4-96* | Chloroflexi | KD4-96 | norank_c__KD4-96 | norank_o__norank_c__KD4-96 | *norank_f__norank_o__norank_c__KD4-96* |
| *uncultured_actinobacterium_g__norank_f__norank_o__norank_c__MB-A2-108* | Actinobacteriota | MB-A2-108 | norank_c__MB-A2-108 | norank_o__norank_c__MB-A2-108 | *norank_f__norank_o__norank_c__MB-A2-108* |
| *uncultured_bacterium_g__Roseiarcus* | Proteobacteria | Alphaproteobacteria | Rhizobiales | Beijerinckiaceae | *Roseiarcus* |
| *unclassified_g__Arthrobacter* | Actinobacteriota | Actinobacteria | Micrococcales | Micrococcaceae | *Arthrobacter* |
| *uncultured_forest_soil_bacterium_g__norank_f__norank_o__Acidobacteriales* | Acidobacteriota | Acidobacteriae | Acidobacteriales | norank_o__Acidobacteriales | *norank_f__norank_o__Acidobacteriales* |
| *unclassified_g__Candidatus_Xiphinematobacter* | Verrucomicrobiota | Verrucomicrobiae | Chthoniobacterales | Xiphinematobacteraceae | *Candidatus_Xiphinematobacter* |
| *unclassified_g__norank_f__Gemmataceae* | Planctomycetota | Planctomycetes | Gemmatales | Gemmataceae | *norank_f__Gemmataceae* |
| *uncultured_forest_soil_bacterium_g__norank_f__norank_o__norank_c__Acidimicrobiia* | Actinobacteriota | Acidimicrobiia | norank_c__Acidimicrobiia | norank_o__norank_c__Acidimicrobiia | *norank_f__norank_o__norank_c__Acidimicrobiia* |
| *uncultured_Acidobacteria_bacterium_g__Occallatibacter* | Acidobacteriota | Acidobacteriae | Acidobacteriales | Acidobacteriaceae_Subgroup_1 | *Occallatibacter* |
| *unclassified_g__Hyphomicrobium* | Proteobacteria | Alphaproteobacteria | Rhizobiales | Hyphomicrobiaceae | *Hyphomicrobium* |
| *metagenome_g__norank_f__TRA3-20* | Proteobacteria | Gammaproteobacteria | Burkholderiales | TRA3-20 | *norank_f__TRA3-20* |
| *uncultured_bacterium_g__Oryzihumus* | Actinobacteriota | Actinobacteria | Micrococcales | Intrasporangiaceae | *Oryzihumus* |
| *unclassified_g__Conexibacter* | Actinobacteriota | Thermoleophilia | Solirubrobacterales | Solirubrobacteraceae | *Conexibacter* |
| *unclassified_g__norank_f__norank_o__Vicinamibacterales* | Acidobacteriota | Vicinamibacteria | Vicinamibacterales | norank_o__Vicinamibacterales | *norank_f__norank_o__Vicinamibacterales* |
| *uncultured_Gemmatimonadales_bacterium_g__norank_f__Gemmatimonadaceae* | Gemmatimonadota | Gemmatimonadetes | Gemmatimonadales | Gemmatimonadaceae | *norank_f__Gemmatimonadaceae* |
| *metagenome_g__norank_f__Acetobacteraceae* | Proteobacteria | Alphaproteobacteria | Acetobacterales | Acetobacteraceae | *norank_f__Acetobacteraceae* |
| *metagenome_g__norank_f__Methyloligellaceae* | Proteobacteria | Alphaproteobacteria | Rhizobiales | Methyloligellaceae | *norank_f__Methyloligellaceae* |
| *uncultured_bacterium_g__Acidothermus* | Actinobacteriota | Actinobacteria | Frankiales | Acidothermaceae | *Acidothermus* |
| *Tomentella_stuposa* | Basidiomycota | Agaricomycetes | Thelephorales | Thelephoraceae | *Tomentella* |
| *uncultured_Acidimicrobiales_bacterium_g__norank_f__norank* | Actinobacteriota | Acidimicrobiia | IMCC26256 | norank_o__IMCC26256 | *norank_f__norank_o__IMCC26256* |
| *unclassified_g__norank_f__norank_o__Subgroup_7* | Acidobacteriota | Holophagae | Subgroup_7 | norank_o__Subgroup_7 | *norank_f__norank_o__Subgroup_7* |
| *Clostridium_estertheticum* | Firmicutes | Clostridia | Clostridiales | Clostridiaceae | *Clostridium_sensu_stricto_13* |
| *unclassified_g__Mycobacterium* | Actinobacteriota | Actinobacteria | Corynebacteriales | Mycobacteriaceae | *Mycobacterium* |
| *unclassified_g__Iamia* | Actinobacteriota | Acidimicrobiia | Microtrichales | Iamiaceae | *Iamia* |
| *unclassified_g__RB41* | Acidobacteriota | Blastocatellia | Pyrinomonadales | Pyrinomonadaceae | *RB41* |
| *Archaeorhizomycetes_sp* | Ascomycota | Archaeorhizomycetes | unclassified_c__Archaeorhizomycetes | unclassified_c__Archaeorhizomycetes | *unclassified_c__Archaeorhizomycetes* |
| *unclassified_f__Mortierellaceae* | Mortierellomycota | Mortierellomycetes | Mortierellales | Mortierellaceae | *unclassified_f__Mortierellaceae* |
| *uncultured_bacterium_g__norank_f__norank_o__Gaiellales* | Actinobacteriota | Thermoleophilia | Gaiellales | norank_o__Gaiellales | *norank_f__norank_o__Gaiellales* |
| *uncultured_Acidimicrobiales_bacterium_g__norank_f__norank* | Actinobacteriota | Acidimicrobiia | IMCC26256 | norank_o__IMCC26256 | *norank_f__norank_o__IMCC26256* |
| *uncultured_Chloroflexi_bacterium_g__norank_f__norank_o__norank_c__AD3* | Chloroflexi | AD3 | norank_c__AD3 | norank_o__norank_c__AD3 | *norank_f__norank_o__norank_c__AD3* |
| *uncultured_Acidobacteria_bacterium_g__norank_f__norank_o__Subgroup_2* | Acidobacteriota | Acidobacteriae | Subgroup_2 | norank_o__Subgroup_2 | *norank_f__norank_o__Subgroup_2* |
| *uncultured_bacterium_g__norank_f__norank_o__JG36-TzT-191* | Proteobacteria | Gammaproteobacteria | JG36-TzT-191 | norank_o__JG36-TzT-191 | *norank_f__norank_o__JG36-TzT-191* |
| *unclassified_g__norank_f__SC-I-84* | Proteobacteria | Gammaproteobacteria | Burkholderiales | SC-I-84 | *norank_f__SC-I-84* |
| *uncultured_Acidobacteria_bacterium_g__Bryobacter* | Acidobacteriota | Acidobacteriae | Bryobacterales | Bryobacteraceae | *Bryobacter* |
| *uncultured_bacterium_g__Aquisphaera* | Planctomycetota | Planctomycetes | Isosphaerales | Isosphaeraceae | *Aquisphaera* |
| *Archaeorhizomycetes_sp* | Ascomycota | Archaeorhizomycetes | unclassified_c__Archaeorhizomycetes | unclassified_c__Archaeorhizomycetes | *unclassified_c__Archaeorhizomycetes* |
| *unclassified_f__Mortierellaceae* | Mortierellomycota | Mortierellomycetes | Mortierellales | Mortierellaceae | *unclassified_f__Mortierellaceae* |
| *uncultured_bacterium_g__norank_f__norank_o__Gaiellales* | Actinobacteriota | Thermoleophilia | Gaiellales | norank_o__Gaiellales | *norank_f__norank_o__Gaiellales* |
| *uncultured_Acidimicrobiales_bacterium_g__norank_f__norank* | Actinobacteriota | Acidimicrobiia | IMCC26256 | norank_o__IMCC26256 | *norank_f__norank_o__IMCC26256* |
| *uncultured_Chloroflexi_bacterium_g__norank_f__norank_o__norank_c__AD3* | Chloroflexi | AD3 | norank_c__AD3 | norank_o__norank_c__AD3 | *norank_f__norank_o__norank_c__AD3* |
| *uncultured_Acidobacteria_bacterium_g__norank_f__norank_o__Subgroup_2* | Acidobacteriota | Acidobacteriae | Subgroup_2 | norank_o__Subgroup_2 | *norank_f__norank_o__Subgroup_2* |
| *uncultured_bacterium_g__norank_f__norank_o__JG36-TzT-191* | Proteobacteria | Gammaproteobacteria | JG36-TzT-191 | norank_o__JG36-TzT-191 | *norank_f__norank_o__JG36-TzT-191* |
| *unclassified_g__norank_f__SC-I-84* | Proteobacteria | Gammaproteobacteria | Burkholderiales | SC-I-84 | *norank_f__SC-I-84* |
| *uncultured_Acidobacteria_bacterium_g__Bryobacter* | Acidobacteriota | Acidobacteriae | Bryobacterales | Bryobacteraceae | *Bryobacter* |
| *uncultured_bacterium_g__Aquisphaera* | Planctomycetota | Planctomycetes | Isosphaerales | Isosphaeraceae | *Aquisphaera* |
| **3500, 4300 m** |  |  |  |  |  |
| *unclassified_o__Acidobacteriales* | Acidobacteriota | Acidobacteriae | Acidobacteriales | unclassified_o__Acidobacteriales | *unclassified_o__Acidobacteriales* |
| *unclassified_f__Micromonosporaceae* | Actinobacteriota | Actinobacteria | Micromonosporales | Micromonosporaceae | *unclassified_f__Micromonosporaceae* |
| *bacterium_Ellin6089* | Proteobacteria | Alphaproteobacteria | norank_c__Alphaproteobacteria | norank_o__norank_c__Alphaproteobacteria | *norank_f__norank_o__norank_c__Alphaproteobacteria* |
| *uncultured_bacterium_g__norank_f__Ilumatobacteraceae* | Actinobacteriota | Acidimicrobiia | Microtrichales | Ilumatobacteraceae | *norank_f__Ilumatobacteraceae* |
| *unclassified_g__norank_f__A0839* | Proteobacteria | Alphaproteobacteria | Rhizobiales | A0839 | *norank_f__A0839* |
| *unclassified_g__GOUTA6* | Proteobacteria | Gammaproteobacteria | Burkholderiales | Nitrosomonadaceae | *GOUTA6* |
| *uncultured_gamma_proteobacterium_g__Acidibacter* | Proteobacteria | Gammaproteobacteria | Gammaproteobacteria_Incertae_Sedis | unclassified_o__Gammaproteobacteria_Incertae_Sedis | *Acidibacter* |
| *uncultured_bacterium_g__Luedemannella* | Actinobacteriota | Actinobacteria | Micromonosporales | Micromonosporaceae | *Luedemannella* |
| *Ilyonectria_rufa* | Ascomycota | Sordariomycetes | Hypocreales | Nectriaceae | *Ilyonectria* |
| *unclassified_g__norank_f__Gemmatimonadaceae* | Gemmatimonadota | Gemmatimonadetes | Gemmatimonadales | Gemmatimonadaceae | *norank_f__Gemmatimonadaceae* |
| *uncultured_planctomycete_g__norank_f__Gemmataceae* | Planctomycetota | Planctomycetes | Gemmatales | Gemmataceae | *norank_f__Gemmataceae* |
| *uncultured_beta_proteobacterium_g__norank_f__SC-I-84* | Proteobacteria | Gammaproteobacteria | Burkholderiales | SC-I-84 | *norank_f__SC-I-84* |
| *uncultured_bacterium_g__Luedemannella* | Actinobacteriota | Actinobacteria | Micromonosporales | Micromonosporaceae | *Luedemannella* |
| *Ilyonectria_rufa* | Ascomycota | Sordariomycetes | Hypocreales | Nectriaceae | *Ilyonectria* |

**Table S****4.** Neighbors of specie CO_3_^-^ and TN in bacterial (fungal)-soil co-occurrence network

| **Species** | **Target** | **Phylum** | **r_value** | **p_value** |
| --- | --- | --- | --- | --- |
| **Fungi** |  |  |  |  |
| *Clavaria* sp | CO_3_^-^ | Ascomycota | 0.810655413 | 1.40E-05 |
| **Bacteria** |  |  |  |  |
| *uncultured_bacterium_g__norank_f__norank_o__norank_c__KD4-96* | CO_3_^-^ | Chloroflexi | 0.696543 | 0.000683 |
| *metagenome_g__norank_f__norank_o__Gaiellales* | CO_3_^-^ | Actinobacteriota | 0.830623 | 0.000006 |
| *uncultured_forest_soil_bacterium_g__norank_f__norank_o__Subgroup_2* | CO_3_^-^ | Acidobacteriota | -0.717996 | 0.000449 |
| *unclassified_g__norank_f__norank_o__norank_c__KD4-96* | CO_3_^-^ | Chloroflexi | 0.609392 | 0.004676 |
| *uncultured_bacterium_g__Roseiarcus* | CO_3_^-^ | Proteobacteria | -0.60001 | 0.005625 |
| *uncultured_forest_soil_bacterium_g__norank_f__norank_o__norank_c__Acidimicrobiia* | CO_3_^-^ | Actinobacteriota | -0.67241 | 0.001146 |
| *metagenome_g__norank_f__TRA3-20* | CO_3_^-^ | Proteobacteria | 0.61744 | 0.00395 |
| *uncultured_bacterium_g__Acidothermus* | CO_3_^-^ | Actinobacteriota | -0.75286 | 0.000146 |
| *uncultured_planctomycete_g__norank_f__Gemmataceae* | CO_3_^-^ | Planctomycetota | -0.685817 | 0.000859 |
| *uncultured_Acidimicrobiales_bacterium_g__norank_f__norank* | CO_3_^-^ | Actinobacteriota | 0.663023 | 0.001421 |
| *uncultured_forest_soil_bacterium_g__norank_f__norank_o__Acidobacteriales* | TOC | Acidobacteriota | 0.711292 | 0.000517 |
| *uncultured_Acidobacteria_bacterium_g__Occallatibacter* | TOC | Acidobacteriota | 0.64151 | 0.00236 |
| *unclassified_g__Iamia* | TOC | Actinobacteriota | -0.61827 | 0.003894 |
| *uncultured_Acidimicrobiales_bacterium_g__norank_f__norank* | TOC | Actinobacteriota | -0.709951 | 0.000525 |
| *uncultured_bacterium_g__Luedemannella* | TOC | Actinobacteriota | -0.811633 | 0.000012 |
